# Rheumatic Irritation in the Diaphragm and Psoas Muscles

**Published:** 1864-12

**Authors:** N. S. Davis

**Affiliations:** Chicago, Illinois


ARTICLE XLIII.

### RHEUMATIC IRRITATION IN THE DIAPHRAGM AND PSOAS MUSCLES.—CLINICAL CASES, REPORTED TO THE CHICAGO MEDICAL SOCIETY, DEC. 23, 1864.

BY N. S. DAVIS, M.D., Chicago, Illinois.

CASE I. Mr. O. H. T——, aged 55 years, naturally robust; sanguine in temperament; accustomed to moderate exercise; and occasionally affected with slight rheumatic attacks; was suddenly seized, on the morning of Nov. 25, 1864, with very violent pain in the right hypochondriac region, extending from the ninth rib down to a level with the crest of the ilium. He had taken his breakfast as usual, and gone to his place of business, when he was seized with the pain rather suddenly, and it became so violent that he was carried to his house, and when I saw him, about two hours after, the pain had reached such a degree of intensity as to appear unendurable. The patient literally writhed in agony, and yet every motion of the body, and especially of respiration, increased the suffering. The pulse was small, 85 per minute; skin cool and moist; face pale; tongue clean; respiration irregular and suppressed to avoid increase of pain; stomach and bowels quiet; and urinary secretions, as far as the patient had observed, nearly natural. About two or three weeks previously the patient had been attacked very suddenly and severely with a pain in the proximal-joint of the great toe, accompanied by some swelling, and closely resembling rheumatic gout. It subsided so rapidly, however, that in forty-eight hours he returned to his business. The location and severity of the pain, in the present attack, was calculated to raise the question of diagnosis between pleurisy, hepatitis, calculi in the biliary ducts, and neuralgic rheumatism in the diaphragm and lower intercostal muscles. The entire absence of fever; the soft and quiet condition of the pulse; and the absence of pleuritic friction, seemed to negative the idea that either of the two first existed; while the absence of vomiting or gastric disturbance equally militated against the supposition that obstruction of the hepatic ducts existed as the cause of suffering.

The preceding attack of pain and swelling in the toe, and the known predisposition of the patient to rheumatism, caused me to regard the present difficulty as neuralgic rheumatism in the right half of the diaphragm, and perhaps extending to the origin of the psoas muscle of the same side. I directed the affected side to be covered with narcotic fomentations, and gave internally the following:—

℞. Vin. Colchici Sem.,---------------------- ℥j.

Acetum Opii.,---------------------------- ℥j.

Mix, and give half a teaspoonful in water every two hours. Also a powder containing calomel, two grains, nitrate potassa, six grains, and sulphate morphia, one-third of a grain, between the doses of the other medicine. The interval between the doses of both medicines was to be lengthened as soon as the severity of the pain was relieved.

At a visit eight hours later, I found the patient much relieved, and lying quiet in bed. Very slight frebrile reaction had come on; had passed a small quantity of urine of a red color; and was drowsy without real sleep. There was some tenderness to pressure over all the space between the lower edge of the ribs and the superior and anterior part of the ilium; and a renewal of very acute pain, with every attempt either to depress the diaphragm by a full inspiration, or to rotate the body, or flex it on the pelvis. The same remedies were continued at longer intervals, until the following morning, Nov. 26th; when the patient was found free from pain and fever so long as he remained quiet in a recumbent position. But acute soreness was still felt on taking a full inspiration or flexing the body on the pelvis; and the urine remained scanty and high colored. The powders were now discontinued, and two drachms of Rochelle salts, in the form of effervescing draught, ordered to be given every three hours until the bowels were moved. After the bowels had been freely moved, he was directed to take a teaspoonful of the following solution every three hours, namely:

℞. Acetas Potassa, Bicarb. Soda, āā ------ ʒiij.

Tinct. Strammonii,--------------------- ʒj.

Water, ---------------------------------- ℥iij.—Mix.

Nov. 27th, patient quite comfortable and able to move with only a moderate feeling of soreness. Continued the saline solution with the strammonium four times a day. From this time the patient continued to improve, and was able to leave the house on the sixth day after the attack.

CASE II. Mr. H——, aged about 30 years; of sanguine temperament; and usually enjoying excellent health; was attacked, on the 18th of Nov'r, 1864, with symptoms of a mild typhoid fever. It continued about two weeks, when the patient appeared to convalesce. On the night of Dec. 5th, however, he was suddenly attacked with very severe pain, extending from the lower ribs of the right side quite down to the iliac fossa; and so greatly aggravated by the slightest effort to turn or flex the body on the pelvis, that the patient could scarcely be induced to move in any degree from a recumbent, dorsal, position, a little inclined to the right side. There was no tympanites or disturbance of the bowels to indicate disease in the cœcum or ascending colon; neither was there much disturbance of the pulse or fever. The urine was scanty, high colored, and gave an unduly acid reaction.

The patient was treated in substantially the same manner as the first case, and recovered so fully as to leave the house and endure quite a journey at the end of nine days from the attack.

CASE III. Mr. S——, aged 33 years, nervo-bilious temperament, active, and of temperate habits; was attacked rather suddenly, on the 5th of December, 1864, with a dull pain in the left side of the abdomen, which was increased by motions of the body, and by flexing the left thigh upon the abdomen. He remained in this condition until the 8th, when the pain become extremely severe, accompanied by some tenderness to pressure over the left side of the abdomen, and entire inability to move the effected side without the most excruciating suffering. The pain seemed to be chiefly located in the track of the left ureter where it crosses the psoas muscles. Though constant, the pain, as in the two preceding cases, had frequent exacerbations. There was no general febrile action present; the skin natural; pulse 80 per minute, and soft; tongue covered with a thin white coat; bowels natural; and urine scanty and high colored. Although there was no pain extending to the testicles or glans penis, yet the suffering of the patient was referred so directly to the course of the ureter, that I was at first disposed to regard the case as one of gravel or calculi in that duct; and prescribed accordingly. At my next visit, twelve hours after, instead of relief, the pain was equally intense and had extended higher up towards the diaphragm. This, with a more careful examination of the effect produced by such movements as call into action the psoas and iliacus interms muscles, led me to regard the case as similar in nature to the two already detailed. I accordingly prescribed the vin. colchici and acetum opii., alternated with the powders of calomel, nitrate potassa and morphia, as in the first case. On the morning of the 9th, I found him much relieved, while quiet but still suffering acute pain on attempting to turn or flex the body.

The colchicum mixture was continued, but instead of the powders, he was directed to take a saline laxative. In the evening he was still further relieved from pain; the laxative had operated twice; and he was suffering some nausea. On account of this last symptom, all other medicines were discontinued, and the following given instead, viz.:—

| | | |
|---|---|---|
| ℞. | Acetas Potassa, | ʒiij. |
| | Bicarb. Soda, | ʒiij. |
| | Acetas Morph., | 3grs. |
| | Water, | ℥iij. |

Mix, and give a teaspoonful every three hours.

From this time the patient continued to improve and was able to leave his room on the 13th inst.

While the foregoing cases were under treatment, two additional ones came under my care, so similar in all respects that a separate description is not necessary. All these cases occurred between the 15th Nov. and the 8th Dec. It was a period of frequent changes of temperature; cool nights, and excessively damp atmosphere.

During the last six weeks, I have also met with seven cases, in which the chief symptoms were, a hard, *twisting* pain in the abdomen; usually in the umbilical region, and constipation of the bowels. The pain was usually increased at night, and accompanied by some tenderness to pressure. There was also in every case, scanty and high-colored urine, with slightly increased frequency and hardness of the pulse. In some, the tongue was clean, in others, it was covered with a thin, white coat, and the skin was warm and dry. Although the bowels were generally constipated, the operation of physic, instead of affording relief more generally increased the abdominal pains and tenderness.

In three of the cases, after suffering with the abdominal pains as above described, for six or seven days, well-marked sub-acute rheumatic inflammation attacked the shoulders and articulations of the upper extremities, accompanied by simultaneous relief from all pain and tenderness in the abdomen. Regarding these cases as true rheumatic irritation in the muscular coat of the intestines, I treated three of the most severe, first with the wine of calchicum seeds and acetated tincture of opium, equal parts, given in doses of half a drachm every three hours, until the pain was relieved: which was accomplished in from 24 to 36 hours.

The bowels were then moved by a powder composed of calomel five grains, and bicarbonate of soda five grains, followed, if necessary, by two drachms of Rochelle salts. After a fair evacuation of the bowels by such means, the patients were directed the following:—

| | | |
|---|---|---|
| ℞. | Bicarb. Soda, ⎫<br>Acetas Potassa, ⎭ | āā ℨiij. |
| | Acetas Morph., | 3grs. |
| | Aqua, | ℥iij. |

Mix, and give a teaspoonful every three or four hours.

Under the influence of this the recovery of the patients was completed in from three to four days. The remaining four cases alluded to were put upon the use of the saline and morphine solution just mentioned, with a powder at night containing eight grains each of Dover's powder and nitrate of potassa, and two grains of calomel. The abdominal pains were relieved by these remedies in from two to three days, when a laxative was given to open the bowels. This was usually followed by a moderate return of pain and soreness, requiring the use of the saline solution four times a day, for three or four days more, to complete the cure.

I have taken the liberty to call the attention of the Society to the foregoing cases, because they seem to be unusual manifestations of rheumatic irritation, and consequently liable to be misunderstood. I hope the questions of diagnosis and treatment will be discussed freely, by the members present.

